# A quadruple fluorescence quantitative PCR method for the identification of wild strains of african swine fever and gene-deficient strains

**DOI:** 10.1186/s12985-023-02111-1

**Published:** 2023-07-14

**Authors:** Xuezhi Zuo, Guorui Peng, Yingju Xia, Lu Xu, Qizu Zhao, Yuanyuan Zhu, Cheng Wang, Yebing Liu, Junjie Zhao, Haidong Wang, Xingqi Zou

**Affiliations:** 1 College of Veterinary Medicine, Shanxi Agricultural University, Jinzhong, 030801 Shanxi China; 2 China/WOAH Reference Laboratory for Classical Swine Fever, China Institute of Veterinary Drug Control, Beijing, China

**Keywords:** *A137R*, *B646L*, *EP402R*, African swine fever, Quadruple qPCR, *MGF505-3R*

## Abstract

**Background:**

Originating in Africa, African swine fever (ASF) was introduced to China in 2018. This acute and highly virulent infectious disease affects domestic pigs. The World Organization for Animal Health has listed it as a statutory reportable disease, and China has listed it as a category A infectious disease.

**Methods:**

Primers and probes were designed for four ASFV genes (*B646L*, *EP402R*, *MGF505-3R*, and *A137R*). The primers/probes were highly conserved compared with the gene sequences of 21 ASFV strains.

**Results:**

After optimization, the calibration curve showed good linearity (R^2^ > 0.99), the minimum concentration of positive plasmids that could be detected was 50 copies/µL, and the minimum viral load detection limit was 10^2^ HAD_50_/mL. Furthermore, quadruple quantitative polymerase chain reaction (qPCR) with nucleic acids from three porcine-derived DNA viruses and cDNAs from eight RNA viruses did not show amplification curves, indicating that the method was specific. In addition, 1 × 10^6^, 1 × 10^5^, and 1 × 10^4^ copies/µL of mixed plasmids were used for the quadruple qPCR; the coefficient of variation for triplicate determination between groups was < 2%, indicating the method was reproducible.

**Conclusions:**

The results obtained by testing clinical samples containing detectable *EP402R*, *MGF505-3R*, and *A137R* strains with different combinations of gene deletions were as expected. Therefore, the established quadruple qPCR method was validated for the molecular diagnosis of ASF using gene-deleted ASFV strains.

**Supplementary Information:**

The online version contains supplementary material available at 10.1186/s12985-023-02111-1.

## Introduction

African swine fever (ASF) is a highly contagious viral disease caused by the African swine fever virus (ASFV), a double-stranded DNA virus belonging to the *Asfarviridae* family, which has 24 known genotypes [1, 2]. Its genome ranges between 170 and 193 kbp in length and encodes 68 structural proteins and > 100 non-structural proteins [3]. The virus comprises four layers of protein shells and an endogenous genome with a significantly more complex structure than many other viruses. In addition, its multilayered structure plays an important role in its replication and survival [4].

The p72 protein encoded by the B646L gene of ASFV is a major coat protein expressed at a late stage with a differential sequence in the C-terminal region [3]. Thus far, ASFV has been classified into 24 genotypes based on partial sequencing of the *B646L* gene encoding p72 [1, 2]. The main strains prevalent in China are genotypes II (reported in 2018) [5] and I (reported in 2021) [6]. In addition, the *EP402R* gene encodes a late expressed CD2v protein, a glycoprotein similar to the surface adhesion receptor CD2v on T lymphocytes [7].Viral CD2v protein is involved in the adsorption of erythrocytes, the binding of extracellular virus particles to erythrocytes [7], host immune regulation, virulence, and induction of protective immune responses [8]. Multigene family (MGF) proteins are widely distributed in ASFV and are generally classified into five families: MGF-100, MGF-110, MGF-300, MGF-360, and MGF-505 [9]. MGF proteins are reported to be early expressed proteins [10] and are key players in multiple stages of transcription, translation, virulence, and immune escape in virally infected host cells [11]. The A137R protein is expressed late during the viral replication cycle, inhibits the interferon signaling pathway, and plays an important role in evading the innate immune response [12]. In the artificial construction of gene-deleted strains, genes such as *EP402R, MGF*, and *A137R* are usually targeted; therefore, establishing corresponding detection methods is necessary for clinical applications.

The diagnosis of ASFV involves a virus isolation–erythrocyte adsorption assay (HAD), polymerase chain reaction (PCR), real-time fluorescent quantitative PCR (qPCR), and isothermal amplification techniques. Virus isolation is a confirmatory method, and its corresponding assay (the erythrocyte adsorption assay) is time-consuming and can be used only to validate strains with erythrocyte adsorption characteristics. Moreover, it must be conducted in a biosafety level III laboratory to measure viral activity in samples and is dependent on the presence of actively replicating virus, which may be absent if the sample has not been correctly stored, resulting in inactivation, thus limiting its clinical applications [13]. The isothermal amplification technique is suitable for rapid on-site detection. However, its sensitivity is slightly less than that of fluorescent PCR. Furthermore, although the PCR method has good specificity, its sensitivity is relatively low, the procedure is cumbersome, and aerosol contamination can easily occur, limiting its applications [14]. However, fluorescent PCR, which has high sensitivity, good specificity, and a convenient procedure, is gradually becoming the main method for ASFV diagnosis. In this technique, the highly sensitive qPCR is the standard method [15].

Because using multiple methods and experiments to detect multiple genes is time-consuming and laborious, only a few genes have been detected using the currently available qPCR methods. In this study, we designed primers/probes for four ASFV genes (*B646L*, *EP402R*, *MGF505-3R*, and *A137R*) and established a quadruple fluorescent qPCR assay to diagnose ASF and differentiate gene-deleted strains from wild-type strains.

## Materials and methods

### Design of primers and probes

Primers/probes for amplifying *B646L*, *EP402R*, *A137R*, and *MGF505-3R* were designed using Primer3 (https://primer3.org/) and were subjected to BLAST analysis. The primers/probes were compared with several ASFV strains published in GenBank using SnapGene software (www.snapgene.com/). The primers/probes (Table 1) were all synthesized by Sangon Biotech (Shanghai, China).The sequences and sizes of the target fragments amplified by the designed primer probes in the Georgia 2007/1 strain are shown in Supplementary file 1, Figs. 1, 2, 3 and 4.

**Table 1:** Primer/probe sequences

Target gene	Primer/probe	Sequences (5'–3')
*B646L*	B646L-F	GAACGTGAACCTTGCTA
	B646L-R	GGAAATTCATTCACCAAATCC
	B646L-P	**6-FAM**-TAAAGCTTGCATCGCA-**MGB**
*EP402R*	EP402R-F	GACACCACTTCCATACATGAAC
	EP402R-R	GGACGCATGTAGTAAATAGGT
	EP402R-P	**Cy5**-CAGTCGTTATCAGTATAA-**MGB**
*A137R*	A137R-F	CTTGAAATCCCTGAGGAACG
	A137R-R	CGATGTCCCGAAATGAGTCT
	A137R-P	**Texas Red**-CACCGCCTGGCATGA-**MGB**
*MGF505-3R*	MGF505-3R-F	GAGCTGTTGTTGTCATGGGA
	MGF505-3R-R	GGATTTTGAATCAGCGGCAA
	MGF505-3R-P	**VIC**-CCCCGCTACGCCGTCGTAGGAGCCC-**MGB**

**Fig. 1:**
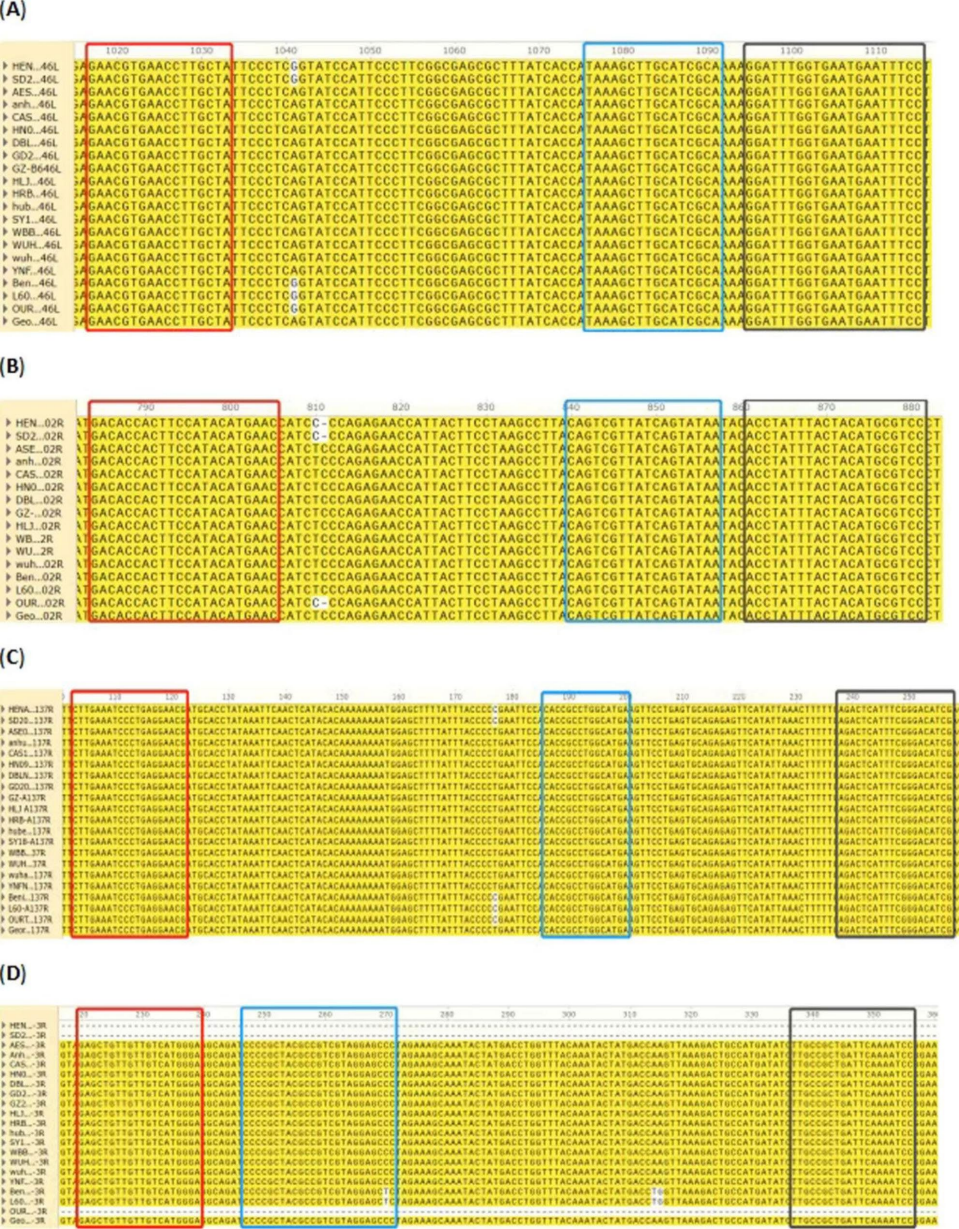
**(A–D)** The primers/probes of *B646L***(A)**, *EP402R***(B)**, *A137R***(C)**, and *MGF505-3R***(D)** with the gene sequence comparison of African swine fever virus (ASFV) endemic strains in China and other countries. The red boxes correspond to the sequence for upstream primers, the blue boxes to the sequence for probes, and the black boxes to the sequence for downstream primers

### Plasmid construction and nucleic acid extraction

With reference to the ASFV HuB20 strain (GenBank sequence number: MW521382), full-length *B646L*, *EP402R*, *A137R*, and *MGF505-3R* genes were synthesized, ligated into the pUC57 vector, and used as a positive control. The recombinant plasmids were named pUC57-B646L, pUC57-EP402R, pUC57-A137R, and pUC57-MGF505-3R; their concentrations were converted to copy numbers after measuring their OD_260_ using a Nanodrop-1000 microspectrophotometer (Thermo Fisher Scientific; Waltham, MA, USA). The four recombinant plasmids were mixed so that the concentration of each recombinant plasmid was 2.5 × 10^8^ copies/µL. Next, the mixed plasmids were diluted to 1 × 10^8^ copies/µL, and 10-fold dilutions were performed to obtain 1 × 10^0^ copies/µL; 1 × 10^2^ copies/µL of the mixed plasmids was twice diluted in half to obtain 25 copies/µL. Lastly, clinical and diluted samples from the 10^8^–10^0^ HAD_50_ ASFV blood series were subjected to nucleic acid extraction using the QIAamp DNA Mini Kit (Cat No. 51,306; Qiagen, Hilden, Germany).

### Quadruple fluorescence quantitative PCR method optimization

Using a LightCycler 480II fluorescent qPCR instrument (Roche Holding AG, Basel, Switzerland), 2× HyperProbe Mixture (CWBIO, Cat No. CW3003M, Beijing, China) was selected as the premix required for the reaction, and the primer/probe concentration, annealing temperature, and cycle number were optimized. Next, the total system (Table 2) was optimized to 25 µL. Predenaturation at 95 °C for 30 s, denaturation at 95 °C for 10 s, and annealing/extension at 58 °C for 20 s were the qPCR reaction conditions. Because of interference between the fluorescence channels, the color compensation procedure was 95 °C for 30 s, 65 °C for 1 min, and 85 °C continuous.

**Table 2:** Quadruple quantitative polymerase chain reaction mixture components

Reagents	Volume (µL)
2× HyperProbe Mixture	12.5
B646L-F (20 µM)	0.3
B646L-R (20 µM)	0.3
B646L-P (10 µM)	0.2
EP402R-F (20 µM)	0.2
EP402R-R (20 µM)	0.2
EP402R-P (10 µM)	0.4
A137R-F (10 µM)	0.4
A137R-R (10 µM)	0.4
A137R-P (10 µM)	0.6
MGF505-3R-F (10 µM)	0.2
MGF505-3R-R (10 µM)	0.2
MGF505-3R-P (10 µM)	0.1
DNA	3
ddH_2_O	6
Total	25

ddH_2_O, double-distilled water

### Establishment of a standard curve

A 10-fold serial dilution of 1 × 10^6^ copies/µL of the mixed plasmid to 10^2^ copies/µL was used as a template for quadruple qPCR amplification. Based on the cycle threshold (Ct) and copy number of the template, a standard curve was generated, and its slope and coefficient of determination (R^2^) were determined.

Sensitivity.

Mixed plasmids of 1 × 10^3^, 1 × 10^2^, 50, 25, and 1 copies/µL were used as reaction templates to test the sensitivity of the quadruple qPCR. In brief, blood samples with a viral load of 10^8^ HAD_50_ ASFV were diluted 10-fold to 10^0^ HAD_50_, and nucleic acids were extracted to test the sensitivity of quadruple qPCR for detecting nucleic acid templates representing different viral loads.

### Specificity and reproducibility

The specificity of the quadruple qPCR was determined using nucleic acids of foot and mouth disease virus (FMDV), bovine viral diarrhea virus (BVDV), porcine epidemic diarrhea virus (PEDV), pseudorabies virus (PRV), porcine parvovirus (PPV), porcine reproductive and respiratory syndrome virus (PRRSV), swine influenza virus (SIV), porcine circovirus II (PCVII), Japanese encephalitis virus (JEV), classical swine fever virus (CSFV), transmissible gastroenteritis virus (TGEV), and ASFV kept in the WOAH reference laboratory of the China Veterinary Drug Inspection Institute. In addition, 1 × 10^6^ to 1 × 10^4^ copies/µL of mixed plasmids were used as templates for triplicate determinations, performed within and between groups of mixed plasmids of each gradient, to test the reproducibility of the quadruple qPCR. The standard deviation and coefficient of variation were calculated.

### Clinical sample testing

The clinical samples collected included blood, liver, spleen, Hubei/2019 genotype II ASFV lung, Genotype 1 ASFV cell samples, and Artificial construction ASFV ΔA137RΔEP402R cell sample. Nucleic acids were extracted from these six samples (200 µL each) and were eluted with 50 µL of eluent, of which 3 µL each was used for the quadruple qPCR. The total system, with each primer/probe, ddH_2_O, and 2× HyperProbe Mixture is presented in Table 2. The reaction conditions are described in Sect. 2.3.

### Declarations

All treatments for viruses were performed in a Biosafety Level III Laboratory of the China Veterinary Drug Inspection Institute.

## Results

### Primer probe design

The gene sequence comparison of the prevalent ASFV strains in China and other countries revealed that the designed primers/probes matched conserved regions in *B646L*, *EP402R*, and *A137R* of ASFV genotypes I and II. In addition, the *MGF505-3R* primers/probes were conserved in genotype I Benin 97/1 (AM712239), genotype I OURT 88/3 (NC_044957), HeN/ZZ-P1/2021 (MZ945536), and SD/DY-I/2021 (MZ945537), and in genotype II strain L60 (KM262844) (Fig. 1 and Supplementary file 2). Therefore, we inferred that the *B646L* primer/probe could be used to confirm ASFV, after which the primers/probes of *EP402R*, *A137R*, and *MGF505-3R* were used to distinguish between the wild-type and gene-deleted ASFV strains.

### Standard curves

**Fig. 2:**
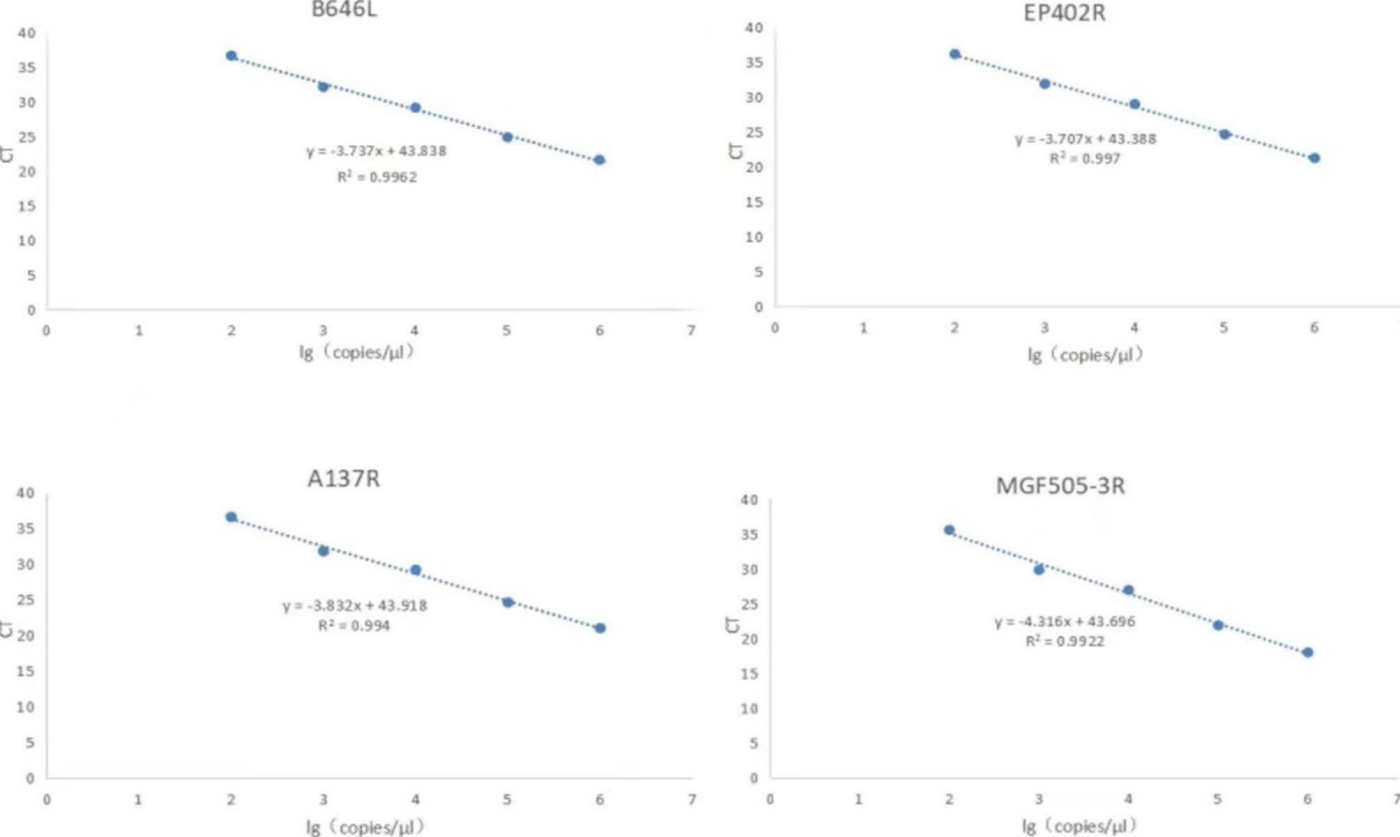
Plot of standard curve equations for *B646L*, *EP402R*, *A137R*, and *MGF505-3R* genes in the quadruple quantitative polymerase chain reaction. *B646L*: y = -3.737x + 43.838, R^2^ = 0.9962. *EP402R*: y = -3.707 + 43.388, R^2^ = 0.997. *A137R*: y = -3.832x + 43.918, R^2^ = 0.994. *MGF505-3R*: y = -4.316x + 43.696, R^2^ = 0.9922

The standard curves obtained using 1 × 10^6^ to 1 × 10^2^ copies/µL of mixed plasmids as templates demonstrated good linearity. Moreover, the slopes of the standard curve equations for *B646L*, *EP402R, A137R*, and *MGF505-3R* were − 3.737, -3.707, -3.832, and − 4.316, respectively; the coefficients of determination (R^2^) were 0.9962, 0.9970, 0.9940, and 0.9922, respectively (Fig. 2 and Supplementary file 3 Figs. 1, 2, 3 and 4). This data indicates that the amplification efficiency of the method was good, and the fit was excellent.

### Minimum detection limit and result determination

Among the mixed plasmids of 1 × 10^3^, 1 × 10^2^, 50 × 10^1^, 25 × 10^1^, and 1 × 10^1^ copies/µL, the minimum limit of detection was 50 × 10^1^ copies/µL for *B646L*, *EP402R*, *A137R*, and *MGF505-3R*, while their Ct values were 39.22, 39.19, 39.76, and 37.30, respectively (Table 3). Furthermore, the minimum limit of detection of 10^2^ HAD_50_/mL was determined using a 10-fold serial dilution of 10^8^ HAD_50_ ASFV blood samples. The Ct values of *B646L*, *EP402R*, *A137R*, and *MGF505-3R* in 10^2^ HAD_50_ nucleic acids were 39.62, 37.93, 38.13, and 35.92, respectively (Table 4).

**Table 3:** Cycle threshold values of plasmids with different copy numbers for each gene

	*B646L*			*EP402R*			*A137R*			*MGF505-3R*		
	Repeat	Mean	SD	Repeat	Mean	SD	Repeat	Mean	SD	Repeat	Mean	SD
1 × 10^3^ copies/µL	32.55	32.51	0.12	33.59	33.10	0.42	33.98	33.63	0.33	30.53	30.35	0.37
	32.38			32.86			33.60			30.60		
	32.60			32.85			33.32			29.92		
1 × 10^2^ copies/µL	37.34	37.03	0.27	38.34	38.35	0.22	37.69	37.61	0.33	36.73	36.13	0.78
	36.93			38.14			37.89			36.41		
	36.83			38.58			37.25			35.25		
50 copies/µL	39.55	39.22	0.30	38.42	39.19	0.67	39.90	39.76	0.40	36.97	37.30	0.60
	39.15			39.51			39.31			36.94		
	38.95			39.64			40.09			38.00		
25 copies/µL												

SD, standard deviation.

**Table 4:** Cycle threshold values of nucleic acids with different viral loads for each gene

	*B646L*			*EP402R*			*A137R*			*MGF505-3R*		
	Repeat	Mean	SD	Repeat	Mean	SD	Repeat	Mean	SD	Repeat	Mean	SD
10^7^ HAD_50_	22.90	22.77	0.27	22.95	22.83	0.19	21.80	21.61	0.28	18.48	18.09	0.35
	22.46			22.62			21.28			17.98		
	22.95			22.94			21.74			17.80		
10^6^ HAD_50_	25.60	25.59	0.06	25.47	25.50	0.21	24.42	24.43	0.30	21.23	21.41	0.27
	25.53			25.31			24.13			21.26		
	25.64			25.72			24.73			21.72		
10^5^ HAD_50_	28.72	28.62	0.09	28.65	28.73	0.10	27.74	27.82	0.12	24.90	25.05	0.20
	28.54			28.69			27.77			24.96		
	28.59			28.84			27.95			25.28		
10^4^ HAD_50_	31.93	31.89	0.18	32.01	32.15	0.24	31.44	31.27	0.41	27.90	28.49	0.52
	31.70			32.01			30.80			28.78		
	32.05			32.43			31.57			28.79		
10^3^ HAD_50_	35.71	35.17	0.47	34.40	34.87	0.49	34.73	34.25	0.49	32.32	32.18	0.26
	34.90			34.82			33.75			31.89		
	34.89			35.39			34.28			32.35		
10^2^ HAD_50_	41.07	39.62	1.27	37.47	37.93	0.47	39.10	38.13	1.12	33.92	35.92	2.34
	38.68			37.91			36.91			38.49		
	39.11			38.41			38.39			35.34		
10^1^ HAD_50_												
10^0^ HAD_50_												

HAD, virus isolation–erythrocyte adsorption assay; SD, standard deviation.

The criteria for determining negative and positive results were based on the results for known low viral load, low copy-number positive plasmid samples, and sensitivity. For *B646L*, a Ct ≤ 37 was considered positive, and Ct > 40 was considered negative. For *EP402R*, a Ct ≤ 38 was considered positive, and Ct > 40 was considered negative. For *A137R*, a Ct ≤ 37 was considered positive, and Ct > 40 was considered negative. For *MGF505-3R*, a Ct ≤ 36 was considered positive, and Ct > 38 was considered negative. For each gene, no Ct value was considered negative, and values between the positive and negative cut-offs were considered suspicious.

### Specificity and reproducibility of the experimental results

**Fig. 3:**
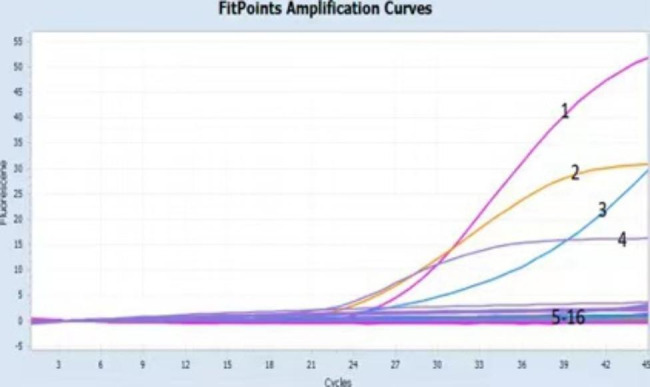
Curves 1–4 represent *B646L*, *MGF505-3R*, *A137R*, and *ER402R* gene amplification profiles of African swine fever virus, respectively, and 5–16 represent pseudorabies virus, porcine parvovirus, porcine circovirus II, foot and mouth disease virus, classical swine fever virus, porcine epidemic diarrhea virus, transmissible gastroenteritis virus, swine influenza virus, Japanese encephalitis virus, porcine reproductive and respiratory syndrome virus, bovine viral diarrhea virus, and negative control, respectively

DNA (PRV, PPV, and PCVII) and RNA viruses (FMDV, CSFV, PEDV, TGEV, SIV, JEV, PRRSV, and BVDV) did not show amplification curves in the quadruple qPCR; only the positive control for ASFV showed typical amplification (Fig. 3), indicating that the established method had good specificity.

The 1 × 10^6^ to 1 × 10^4^ copies/µL of mixed plasmids showed good reproducibility in the three replicates between groups within the quadruple qPCR, with coefficients of variation of less than 2% in all cases (Table 5).

**Table 5:** Intra-group reproducibility of the quadruple quantitative polymerase chain reaction groups

Target gene	Concentration (copies/µL)	Intra-group replication			Repeated between groups		
		Mean	SD	CV%	Mean	SD	CV%
*B646L*	1 × 10^6^	21.70	0.05	0.2	21.93	0.21	1
	1 × 10^5^	24.92	0.03	0.1	25.15	0.2	0.8
	1 × 10^4^	29.23	0.06	0.2	29.76	0.47	1.6
*EP402R*	1 × 10^6^	21.23	0.03	0.1	21.43	0.21	1
	1 × 10^5^	24.62	0.06	0.2	24.77	0.19	0.8
	1 × 10^4^	28.97	0.22	0.8	29.40	0.38	1.3
*A137R*	1 × 10^6^	20.97	0.03	0.1	21.24	0.24	1.1
	1 × 10^5^	24.56	0.13	0.5	24.74	0.20	0.8
	1 × 10^4^	29.14	0.23	0.8	29.62	0.42	1.4
*MGF505-3R*	1 × 10^6^	17.97	0.02	0.1	18.31	0.32	1.7
	1 × 10^5^	21.86	0.11	0.5	22.26	0.37	1.7
	1 × 10^4^	26.94	0.20	0.7	27.50	0.52	1.9

SD, standard deviation; CV, coefficient of variation.

### Quadruple qPCR method validation

**Fig. 4:**
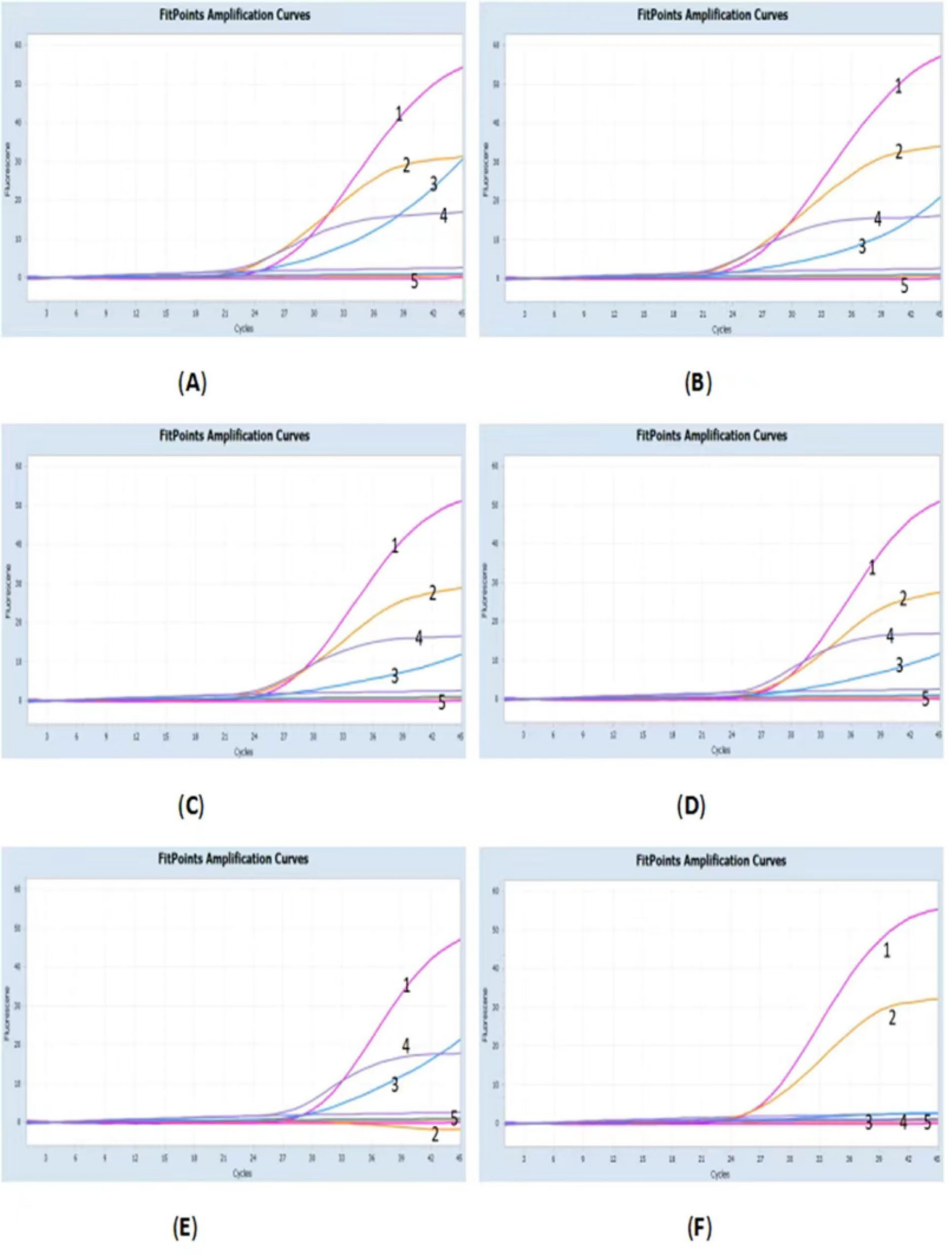
Amplification curves for (**A**) the genotype II African swine fever virus (ASFV) blood sample, (**B**) the genotype II ASFV liver sample, (**C**) the genotype II ASFV spleen sample, (**D**) the genotype II ASFV lung sample, (**E**) the genotype I cell sample, and (**F**) the ASFV ΔA137RΔEP402R cell sample. Numbers 1–5 indicate ASFV *B646L*, *MGF505*-*3R*, *A137R*, *ER402R*, and negative control amplification curves, respectively

The clinical samples tested using the established quadruple qPCR showed the expected amplification. ASFV nucleic acids from the blood, liver, spleen, and lungs showed amplification; however, the *MGF505-3R* of genotype I ASFV and the *A137R* and *EP402R* of ASFVΔA137RΔEP402R did not show amplification (Fig. 4).

## Discussion

Since its discovery, the continuous spread of ASF has significantly affected the global supply of pork products and has devastated food security and animal health and welfare [16]. China’s pig production capacity has decreased significantly since the disease was introduced in 2018. Because of the insidiousness and complexity of ASFV transmission, the epidemic remains unresolved [17]. Although ASF has long been identified, it lacks a safe and effective vaccine. Therefore, an effective diagnostic method is critical for controlling the epidemic. Consequently, we designed primers/probes for four genes, *B646L*, *EP402R*, *A137R*, and *MGF505-3R*. Notably, the p72 protein, the main capsid protein of ASFV encoded by the *B646L* gene, is often used as the first choice for diagnosing epidemic ASFV [18–21]. In addition, CD2v, encoded by the *EP402R* gene, is vital for ASFV diagnosis [22, 23]. Furthermore, *EP402R*, *MGF*, and *A137R* are known virulence genes whose deletion can substantially reduce the virulence of the virus in pigs [23–30]. Therefore, these genes are expected to serve as alternative deletion genes for gene deletion vaccines, and establishing corresponding identification methods is necessary. Moreover, mutant strains such as CD2v-deletion strains with low pathogenicity have previously been identified [31]. We considered that targeting these ASFV genes would be necessary to confirm the diagnosis and pathogenic strains involved in ASFV infection. Thus, we developed a suitable quadruple PCR method that showed high sensitivity and specificity.

The *B646L*, *EP402R*, and *A137R* primers/probes used in this study were conserved in genotypes I and II ASFV. In addition, the *MGF505-3R* primers/probes were conserved in genotype I Benin 97/1 (AM712239), L60 (KM262844), and genotype II ASFV. However, deletions were found in OURT 88/3 (NC_044957) and in the frequently isolated Chinese genotypes HeN/ZZ-P1/2021 (MZ945536) and SD/DY-I/2021 (MZ945537). With this method, we inferred that a sample with positive results for *B646L*, *EP402R*, and *A137R* and negative results for *MGF505*-*3R* could contain genotype I ASFV. Moreover, we verified this result with a known genotype I ASFV cytotoxic sample (see Fig. 4E), and the results were consistent with our hypothesis.

To verify the specificity of the method, we performed amplification using nucleic acids of three porcine-derived DNA viruses (PRV, PPV, and PCVII) and eight porcine-derived RNA viruses (FMDV, CSFV, PEDV, TGEV, SIV, JEV, PRRSV, and BVDV). None of these 11 nucleic acids showed amplification curves; only the positive ASFV control showed typical amplification (see Fig. 3), indicating that the method was specific for diagnosing ASFV without interference from other pathogens. Regarding reproducibility, the coefficient of variation was calculated for triplicate determination within each group, and the results obtained for all four genes were < 2% less than that of other ASFV qPCR diagnostic methods [32]. This result indicated that our method was reproducible, with minimal deviation in the results obtained from each experiment, and that batch differences do not affect the determination of the results.

Regarding sensitivity, the minimum limit of detection for all four genes was 50 copies/µL and 10^2^ HAD_50_/mL. Moreover, our method was more sensitive for the detection of *B646L* and *EP402R* than other qPCR methods [33]. Because of the interference of the fluorescent groups in each probe of the quadruple qPCR, the hydrolysis efficiency of the probes was affected, changing the amplification efficiency. However, these effects were within a reasonable range, and the determination of negative results was unaffected. In addition, the method allowed the simultaneous detection of four genes, shortening the time of multigene detection.

A limitation of our study is that only four genes could be detected as only a maximum of four fluorescence channels are available in the current fluorescence PCR instruments. In the future these instruments may improve to include more fluorescence channels, which would allow for detection of additional genes.

In conclusion, we established a quadruple qPCR method for *B646L*, *EP402R*, *A137R*, and *MGF505*-*3R* to distinguish ASFV wild-type strains from gene-deleted strains based on current research. This method is the only qPCR method that can simultaneously detect four ASFV genes with conserved primer/probe sequences with high sensitivity, specificity, and reproducibility, providing a comprehensive diagnosis of ASFV.

## Electronic supplementary material

Below is the link to the electronic supplementary material.

Supplementary Material 1

Supplementary Material 2

Supplementary Material 3

## Data Availability

All data generated or analysed during this study are included in this published article and its supplementary information files.

## Abbreviations

ASF: African swine fever
ASFV: African swine fever virus
BVDV: Bovine viral diarrhea virus
CSFV: Classical swine fever virus
FMDV: Foot and mouth disease virus
HAD: Virus isolation–erythrocyte adsorption assay
JEV: Japanese encephalitis virus
MGF: Multigene family
PCVII: Porcine epidemic diarrhea virus
PEDV: Porcine epidemic diarrhea virus
PPV: Porcine parvovirus
PRRSV: Porcine reproductive and respiratory syndrome virus
PRV: Pseudorabies virus
qPCR: Quantitative polymerase chain reaction
SIV: Swine influenza virus
TGEV: Transmissible gastroenteritis virus
