# Supplementary material for: A quadruple fluorescence quantitative PCR method for the identification of wild strains of african swine fever and gene-deficient strains
Source: Virol J. 2023 Jul 14;20:150. doi: 10.1186/s12985-023-02111-1 (PMC10347796; doi:10.1186/s12985-023-02111-1)
Supplement: Supplementary file 1 — Supplementary Material 1 [file 12985_2023_2111_MOESM1_ESM.docx]

|  | GenBank | Name |
| --- | --- | --- |
| genotype I ASFV | MZ945536 | HeN/ZZ-P1/2021 |
|  | MZ945537 | SD/DY-I/2021 |
|  | NC_044957 | OURT 88/3 |
|  | AM712239 | Benin 97/1 |
|  | KM262844 | L60 |
| genotypeⅡASFV | MK940252 | AES01 |
|  | MK128995 | AnhuiXCGQ |
|  | MN172368 | CAS19-01 |
|  | MZ614662 | CADC_HN09 |
|  | MK333181 | DB/LN/2018 |
|  | MW361944 | GD/2019 |
|  | ON263123 | GZ201801_2 |
|  | MK333180.1 | HLJ/2018 |
|  | MW656282 | HRB1/2020 |
|  | MW521382 | HuB20 |
|  | MH766894 | SY18 |
|  | MN393477 | Wuhan 2019-2 |
|  | MK645909 | wbBS01 |
|  | MN393476 | Wuhan 2019-1 |
|  | ON400500 | YNFN202103 |
|  | Georgia 2007/1 | FR682468 |

***Supplementary Material 2***
