# Supplementary material for: A quadruple fluorescence quantitative PCR method for the identification of wild strains of african swine fever and gene-deficient strains
Source: Virol J. 2023 Jul 14;20:150. doi: 10.1186/s12985-023-02111-1 (PMC10347796; doi:10.1186/s12985-023-02111-1)
Supplement: Supplementary file 2 — Supplementary Material 2 [file 12985_2023_2111_MOESM2_ESM.docx]

***Supplementary Material 1***


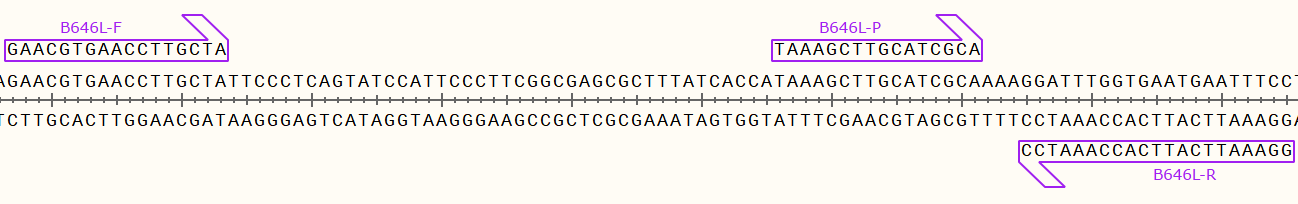


**Figure 1.** Sequence map of the target fragment gene of B646L gene amplified in the Georgia 2007/1 strain, which is 99bp in length.


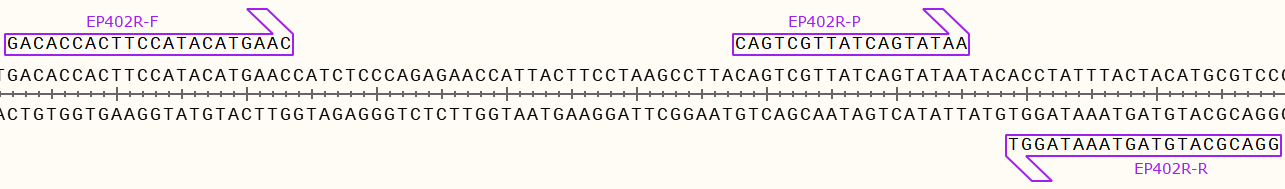


**Figure 2.** Sequence map of the target fragment gene of EP402R gene amplified in the Georgia 2007/1 strain, which is 98bp in length.


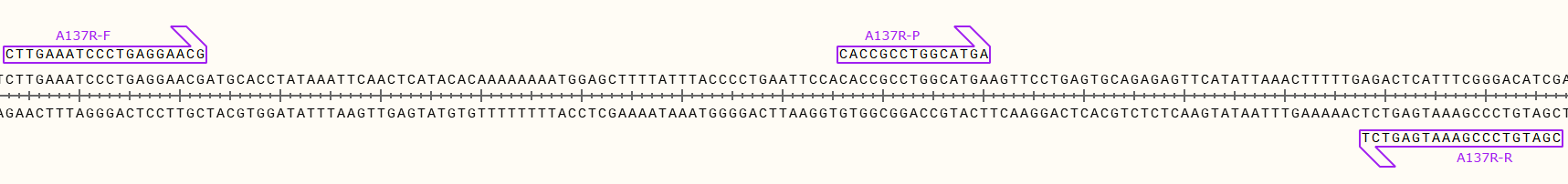


**Figure 3.** Sequence map of the target fragment gene of the A137R gene amplified in the Georgia 2007/1 strain, which is 155 bp in length.


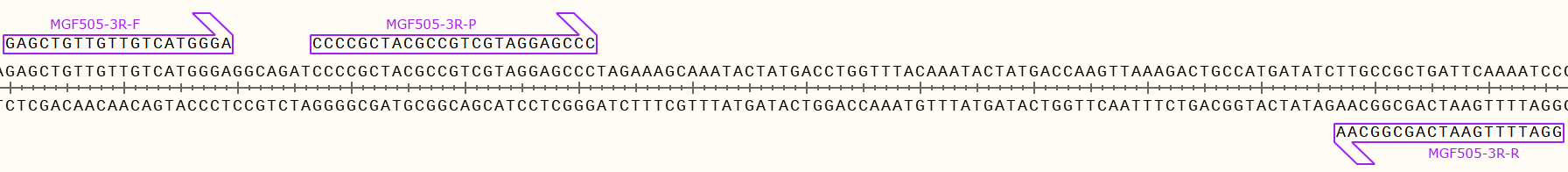


**Figure 4.** Sequence map of the target fragment gene of MGF505-3R gene amplified in the Georgia 2007/1 strain, which is 137bp in length..
