# Supplementary material for: A quadruple fluorescence quantitative PCR method for the identification of wild strains of african swine fever and gene-deficient strains
Source: Virol J. 2023 Jul 14;20:150. doi: 10.1186/s12985-023-02111-1 (PMC10347796; doi:10.1186/s12985-023-02111-1)
Supplement: Supplementary file 3 — Supplementary Material 3 [file 12985_2023_2111_MOESM3_ESM.docx]

Supplementary Material 3


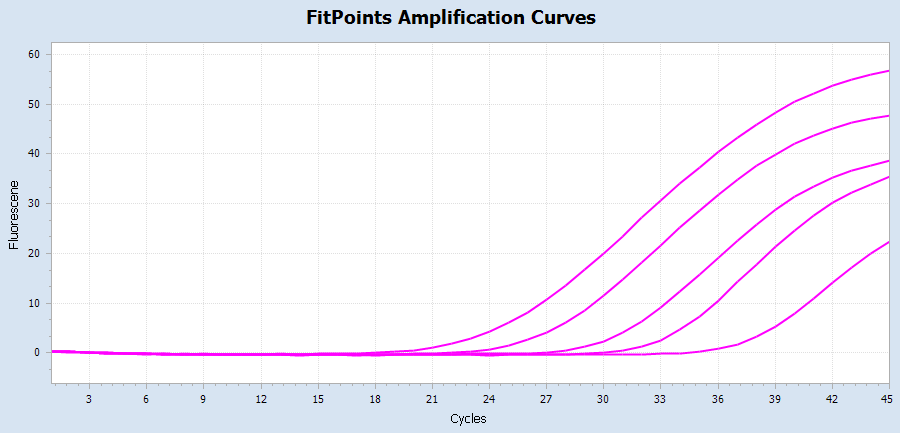


**Figure 1.** *B646L* gene amplification curve. From left to right, the mixed plasmid reaction templates are 10^6^, 10^5^, 10^4^, 10^3^, and 10^2^ copies/μL.


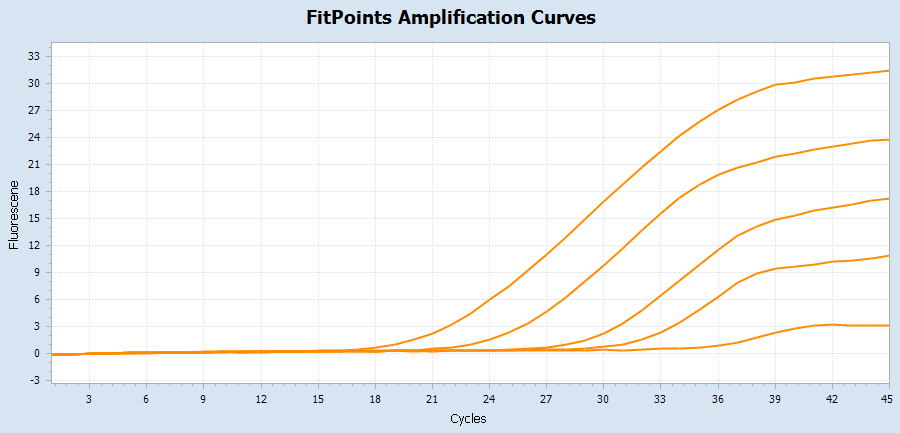


**Figure 2.** *MGF505-3R* gene amplification curve. From left to right, the mixed plasmid reaction templates are 10^6^, 10^5^, 10^4^, 10^3^, and 10^2^ copies/μL.


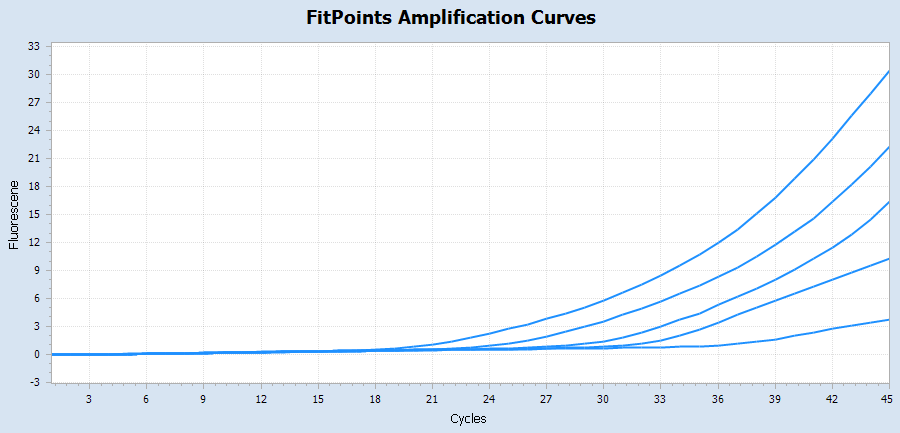


**Figure 3.** *A137R* gene amplification curve. From left to right, the mixed plasmid reaction templates are 10^6^, 10^5^, 10^4^, 10^3^, and 10^2^ copies/μL.


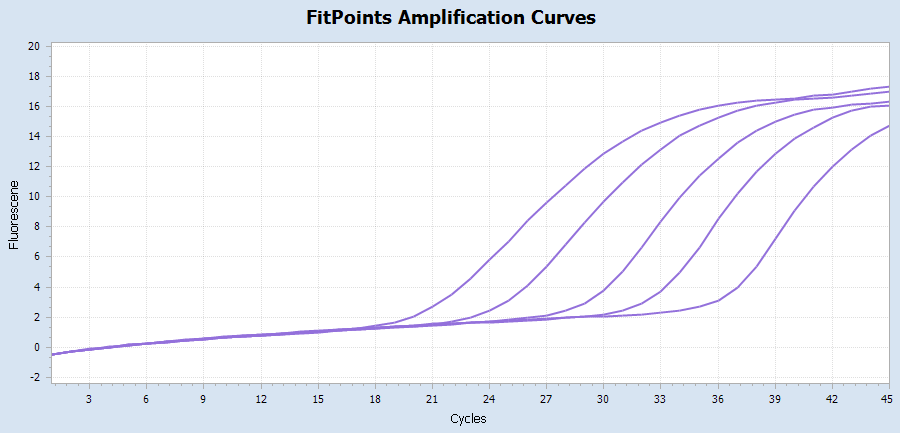


**Figure 4.** *EP402R* gene amplification curve. From left to right, the mixed plasmid reaction templates are 10^6^, 10^5^, 10^4^, 10^3^, and 10^2^ copies/μL.
